# Simultaneous Application of Methylene Blue and Chlorin e6 Photosensitizers: Investigation on a Cell Culture

**DOI:** 10.17691/stm2025.17.1.06

**Published:** 2025-02-28

**Authors:** A.V. Ryabova, I.D. Romanishkin, I.V. Markova, D.V. Pominova

**Affiliations:** Senior Researcher, Laser Biospectroscopy Laboratory, Light-Induced Surface Phenomena Department, Natural Sciences Center; Prokhorov General Physics Institute of the Russian Academy of Sciences, 38 Vavilov St., Moscow, 119991, Russia; Associate Professor, Department 87 “Laser Micro-, Nano-, and Biotechnologies, Engineering Physics Institute for Biomedicine”; National Research Nuclear University MEPhI, 31 Kashirskoye Highway, Moscow, 115409, Russia; Junior Researcher, Laser Biospectroscopy Laboratory, Light-Induced Surface Phenomena Department, Natural Sciences Center; Prokhorov General Physics Institute of the Russian Academy of Sciences, 38 Vavilov St., Moscow, 119991, Russia; PhD Student; National Research Nuclear University MEPhI, 31 Kashirskoye Highway, Moscow, 115409, Russia; Engineer, Laser Biospectroscopy Laboratory, Light-Induced Surface Phenomena Department, Natural Sciences Center; Prokhorov General Physics Institute of the Russian Academy of Sciences, 38 Vavilov St., Moscow, 119991, Russia; PhD, Senior Researcher, Laser Biospectroscopy Laboratory, Light-Induced Surface Phenomena Department, Natural Sciences Center; Prokhorov General Physics Institute of the Russian Academy of Sciences, 38 Vavilov St., Moscow, 119991, Russia; Associate Professor, Department 87 “Laser Micro-, Nano-, and Biotechnologies, Engineering Physics Institute for Biomedicine”; National Research Nuclear University MEPhI, 31 Kashirskoye Highway, Moscow, 115409, Russia

**Keywords:** photodynamic therapy, hypoxia, methylene blue, chlorin e6, oxidative phosphorylation, aerobic glycolysis

## Abstract

The application of photosensitizers for inhibition of oxidative phosphorylation in order to temporally decrease oxygen uptake by tumor cells in the course of photodynamic therapy (PDT) evokes growing interest.

**The aim of the study** is to overcome tumor hypoxia for further photodynamic therapy with simultaneous use of type I photosensitizer methylene blue (MB) and type II photosensitizer chlorin e6.

**Material and Methods:**

A photodynamic activity of MB and its combined use with chlorin e6 has been studied on the HeLa cell culture, their effect on cell metabolism in their co-accumulation and subsequent irradiation has also been assessed.

**Results:**

MB generates reactive oxygen species in the cells in contrast to chlorin e6, which produces singlet oxygen. Besides, MB is converted to a colorless leucoform at low concentrations in the process of de-oxygenation. Incubation of cells with MB concurrently with chlorin e6 results in its greater fluorescence as compared to the incubation with MB only. MB concentration in the range of 1–10 mg/kg and the laser radiation dose of 60 J/cm^2^ do not cause cell death, probably, due to the MB transition to the photodynamically inactive leucoform. Cell death is observed after PDT in all samples with chlorin e6 and with MB at the 0–20 mg/kg concentration ranges and at 60 J/cm^2^ radiation dose. The phototoxicity of MB together with chlorin e6 is higher than that of chlorin e6 alone. The analysis of metabolic NADH cofactor lifetime after the incubation of the cells with MB and chlorin e6, and after PDT with them has revealed the presence of stress seen as an extension of NADH fluorescence cloud along the metabolic axis. After PDT with low concentrations of MB, the NADH fluorescent cloud on the phasor diagram shifts to the right towards short lifetimes (closer to anaerobic glycolysis along the NADH metabolic trajectory). The PDT with MB and chlorin e6 leads to the shift of the NADH fluorescence cloud on the phasor diagram to the left towards long lifetimes (closer to oxidative phosphorylation along the NADH metabolic trajectory). In this case, the cells die due to necrosis.

**Conclusion:**

The co-accumulation of MB with chlorin e6 prevents MB reduction to a colorless leucoform, decreasing the oxygen uptake by the cells and making it possible to use simultaneously type I and II photodynamic reactions.

## Introduction

Tumor microenvironment consists of tumor, immune, and stromal cells, blood vessels, and extracellular matrix and represents a physiological medium different from normal tissues, with a low content of oxygen, lower pH, vascular anomaly, immunosuppressive properties, and higher interstitial pressure [1]. The efficacy of antitumor therapy, photodynamic therapy (PDT) in particular, is inversely related to the degree of hypoxia. Two strategic approaches may be distinguished to overcome hypoxia and to further conduct PDT: to increase oxygen content in the tumor tissue for oxygen-dependent PDT and to use oxygen-dependent PDT with photosensitizers acting by the mechanism of type I photodynamic reactions [2]. In order to increase oxygen concentration in the tumor tissues, the following approaches are considered: its exogenous delivery, *in situ* generation, decreased uptake of oxygen by the tumor cells, and normalization of blood circulation in the tumor [3].

Cell respiration is the main process of oxygen consumption by the living cells. Healthy differentiated cells rely mainly on oxidative phosphorylation (OXPHOS) in mitochondria for receiving energy, whereas tumor cells use aerobic glycolysis instead of OXPHOS even in the medium enriched with oxygen, which is known as the Warburg effect [4]. Simultaneous switching off the energy supplying pathways such as OXPHOS and glycolysis is the most direct strategy of cancer treatment [5, 6]. OXPHOS inhibition may diminish oxygen uptake by the cells and thereby increase its partial pressure, decreasing hypoxia and further enhancing the PDT effect [7, 8]. On the other hand, OXPHOS inhibition promotes enhancement of glycolysis, which provides a fast synthesis of adenosine triphosphate and growth of the tumor. In addition, lactate, which is a byproduct of glycolysis, influences the tumor microenvironment and signaling pathways [9, 10]. More complicated mechanisms of action of clinically available preparations are expected to be discovered for remodeling the microenvironment of tumor hypoxia, which can also facilitate the treatment of tumor hypoxia.

The other approach to overcoming the limitations in the therapy related to hypoxia is the application of photosensitizers using type I mechanism of photodynamic reaction, for example, methylene blue (MB). It is a phenothiazine dye, which is used as antiseptic and antidote. However, the effect of MB on cell metabolism is rather uncommon. MB is a redox agent; in the conditions of acute mitochondrial dysfunction, the redox cyclic properties of MB allow for reactivation of OXPHOS and the Krebs cycle via an alternative route of NADH oxidation [11, 12]. MB accepts electrons from the reducing equivalents in mitochondria and transfers them to other components of the respiration chain or molecular oxygen bypassing the activity of complexes I, II, III of the electron transport chain [13]. Previously, we have shown that MB can cause a positive effect on oxygenation on the mouse model of the reinoculated tumor [14]. However, there are difficulties in working with MB; it is prone to dimer generation at a high concentration and to transition to a colorless leucoform in hypoxia.

In the present work, photodynamic activity of MB and MB in the combination with chlorin e6 has been studied on a cell culture and their effect on cell metabolism in their co-accumulation and subsequent irradiation has been assessed. The idea of concurrent application of type I (MB) and type II (chlorin e6) photosensitizers seems to be interesting owing to their diverse influence on cell metabolism [15].

Chlorin e6 is a type II photosensitizer generating rapidly utilized single oxygen ^1^О_2_. As recent studies show, it causes an inhibiting effect on the tricarboxylic acid cycle, and, consequently, on OXPHOS [16]. Presumably, chlorin e6 inhibits the tricarboxylic acid cycle resulting in the diminished number of the reduced NADH and FADH_2_ coenzymes, in the form of which oxidation energy, released at different stages of the cycle, is stored. At the same time, the rate of MB transition to the colorless photoinactive leucoform decreases due to MB reduction by the interaction with NADH coenzyme. This enhances the effect of type I PDT with MB supplementing the effect from type II PDT with chlorin e6. To confirm this hypothesis, the investigation of PDT with two photosensitizers (MB and chlorin e6) has been carried out on cell cultures.

## Materials and Methods

### Materials

0.35% radachlorin solution (RADAFARMA, Russia) and 1% aqueous solution of methylene blue (Samaramedprom, Russia) were used in our work.

### Cell culture

HeLa cells of epitheloid cervical carcinoma were cultivated in DMEM medium (Gibco, USA) with the addition of 10% FBS (Gibco, USA) and 1% antibiotic/antimycotic solution (penicillin/streptomycin) (Corning, USA) at 37°C and 5% CO_2_. The cells were reseeded using 0.05% trypsin solution (Gibco, USA).

### Investigation of the intracellular production of MB by reactive oxygen species (ROS)

HeLa cells were seeded on the plates for confocal microscopy a day before the experiment; on the day of the experiment, they were incubated with MB at the concentration of 10 mg/kg for 1 h. Then a fluorescent indicator was added and the effect of laser irradiation with 660 nm wavelength at a dose of 30 J/cm^2^ was studied for generation of photochemical reaction products *in vivo* by the fluorescence cell images using the inverted LSM- 710-NLO laser scanning confocal microscope (Carl Zeiss, Germany).

We used ROS (hydrogen peroxide H_2_O_2_, superoxide anion O_2^−^_, hydroxyl radical OH, hydroperoxides ROOH, and peroxy-nitrites ONOO^−^) indicator — 6-carboxy- 2′,7′-dichlorodihydrofluorescein diacetate (6-Carboxy- H_2_DCFDA; Lyumiprob RUS, Russia) at the concentration of 1 μm in the medium incubating it with the cells for 30 min. 6-Carboxy-H_2_DCFDA was excited by the 488 nm laser and fluorescence was detected in the 510–580 nm range.

### Investigation of MB transition to the reduced colorless form under hypoxia conditions during its accumulation in the cells

One day before the experiment, the HeLa cells were seeded in the POCmini-2 Cell Cultivation System (PeCon GmbH, Germany) for confocal microscopy. The cells were cultured in a complete growth medium with subsequent addition of MB at the doses 10 and 100 mg/kg for 4 h. The cells underwent 5-fold washing from the MB medium before measurement of absorption spectra and microscopy. The open cultivation plate with the studied cell monolayer for confocal microscopy was reassembled to obtain a closed cultivation plate with the possibility of perfusion. The final plate assembly consisted of two cover glasses separated by a 2 mm spacer with a cell monolayer on one of the glasses. The plate was purged with nitrogen for 10 min. This time duration was established to be sufficient for complete de-oxygenation of the media on the specimens with erythrocytes, which was validated spectroscopically by the spectra of erythrocyte hemoglobin absorption.

Absorption spectra of the HeLa monolayer before and after incubation with MB, before and after purging with nitrogen were recorded using Hitachi U3400 spectrophotometer (Hitachi, Japan) in the 350–900 nm range. Fluorescence lifetime imaging microscopy (FLIM) was used to study the changes in cell respiration based on NADH autofluorescence and to observe the changes of the MB fluorescence lifetime.

### Investigation of NADH and MB fluorescence lifetime

FLIM was used to analyze the type of cell respiration based on NADH fluorescence lifetime and MB aggregation level. Cell autofluorescence was recorded at two-photon excitation at the 740 nm wavelength with the femtosecond Chameleon Ultra II laser (Coherent, USA) using the FLIM module (Becker & Hickl GmbH, Germany) connected to the LSM-710-NLO microscope (Carl Zeiss, Germany). The FLIM module consisted of the time-correlated SPC-150 system for single photon counting, hybrid GaAsP HPM100-07 photodetector, and SPCM software. The Plan-Apochromate 63x/1.4 Oil objective (Carl Zeiss, Germany) was used. The FB450-40 band filter (Thorlabs, USA) was employed to isolate the fluorescence signals from NADH. The timeresolved fluorescence images were processed using the SPCImage 8.0 program (Becker & Hickl GmbH, Germany). The time-resolved fluorescence data were processed by the method of vector diagrams, where the fluorescence lifetime is indicated in a frequency presentation [17]. The KernelDensity and Makie libraries for Julia programming language were employed to build vector diagrams.

### Intracellular accumulation of MB and chlorin e6

The solution of MB or MB with chlorin e6 was added to the culture medium to reach the concentration of 1, 5, 10, and 20 mg/kg and 5 mg/kg of chlorin e6, then incubated at 37°C, 5% CO_2_ for an hour. The intensity of photosensitizer fluorescence in the culture was analyzed using confocal laser scanning microscopy. MB and chlorin e6 were excited at 633 nm wavelength, fluorescence was recorded in the 645–750 nm spectral range.

### Investigation of the photodynamic effect of MB and chlorin e6 on the cells

The solution of MB or MB with chlorin e6 was added to the culture medium to reach the concentration of 1, 5, 10, and 20 mg/kg and 5 mg/kg of chlorin e6, then incubated at 37°C, 5% CO_2_ for an hour. Photodynamic exposure was performed by excitation with 660 nm laser; power density was 100 mW/ cm^2^. The irradiation lasted for 10 min, the total light dose was equal to 60 J/cm^2^. Fluorescence intensity in the cells was assessed before, immediately after irradiation and the next day after irradiation using confocal laser scanning microscopy. The day after irradiation, staining with acridine orange and propidium iodide (AO/PI) was done to visualize the living/dead cells.

## Results

### Photochemical properties of MB in the cells

**Figure 1.**
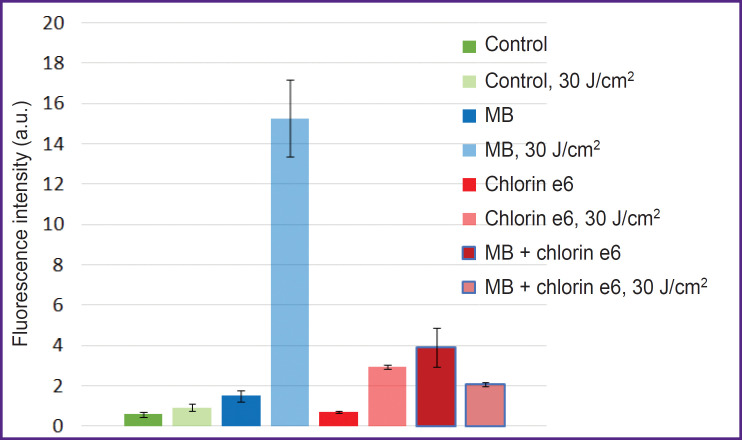
Generation of ROS in the HeLa cell culture before and after 30 J/cm^2^ irradiation

Investigations with ROS indicator 6-Carboxy-H_2_DCFDA have found that the addition of MB to the cells results in effective ROS generation. Moreover, PDT with MB leads to more intensive MB fluorescence inside the cells (Figure 1).

The ROS generation rate in case of dark MB accumulation, especially in the presence of chlorin e6, is considerable. It is likely related to the involvement of MB in the electron transport chain, whose complex I and II generates mitochondrial ROS [18]. The property of MB in the cells to generate ROS is confirmed by the data of the study [19], which demonstrated the liability of MB to photodynamic type I reactions including the electron transfer and formation of semi-reduced and semi-oxidized MB radicals resulting in further oxidation of biomolecules.

### Generation of MB aggregates

At large MB concentrations, there occurs generation of aggregates leading to the changes in membrane fluidity, including mitochondrial membranes, and to impairment of mitochondrial potential. According to the literature data, aggregation is not significant at the MB concentration in water below 3.4×10^−5^ M (11.03 mg/kg), whereas at the concentration higher than 2.09×10^−4^ M (66.85 mg/kg) dimer assembly takes place [20]. To study the effect of aggregation, we used 10 and 100 mg/kg MB concentrations, assuming that in the first case the dimer concentration is lower, while in the second case it is higher.

**Figure 2.**
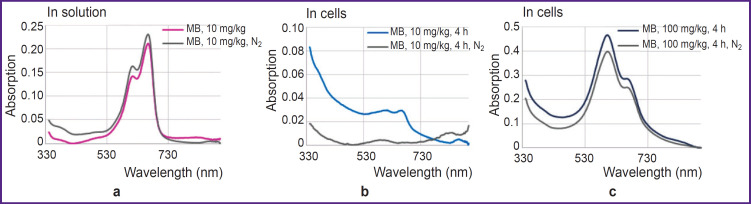
Absorption spectra: (а) for the MB solution at the concentration of 10 mg/kg before and after nitrogen purging; (b) and (c) for the HeLa cell monolayer after incubation with MB at the concentration of 10 and 100 mg/kg, respectively, before and after nitrogen purging

**Figure 3.**
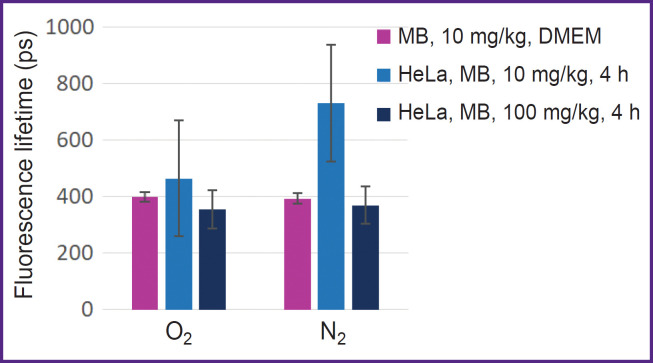
Average lifetime of MB and HeLa fluorescence after incubation with MB at the concentration of 10 and 100 mg/kg in the cells before and after nitrogen purging

Absorption spectra of MB solution at the 10 mg/kg concentration and HeLa monolayer after incubation with MB at the concentrations of 10 and 100 mg/kg before and after nitrogen purging are presented in Figure 2 (a), (b), (c), respectively, and fluorescence lifetime is shown in Figure 3.

At low MB concentrations and de-oxygenation in the cells, MB changes to the colorless leucoform. The discoloration effect is not observed at high MB concentrations. It may be because dimers of MB are not involved in the redox reactions of the cell and, therefore, no transition to the leucoform occurs. Besides, a high concentration of MB as a redox cycler may lead to proton depletion, which also impacts this process.

MB fluorescence lifetime in the solution and inside the cells differs only for the 10 mg/kg concentration under the conditions of normal gas circulation and in hypoxia. According to the literature data [21], fluorescence lifetime for MB monomers is ~370 ps, while for MB dimer it is below 20 ps. Despite the fact that during nitrogen purging the peak of MB monomer absorption in the cells after incubation with 10 mg/kg MB has actually disappeared, MB fluorescence remained and its lifetime has grown from 400 to 700 ps.

### The results of cell incubation with MB and chlorin e6

To study simultaneous accumulation of MB and chlorin e6, the cells were incubated in the complete medium with MB at the concentration of 1, 5, 10, and 20 mg/kg without and with chlorin e6 at the concentration of 5 mg/kg at 37°C, 5% CO_2_ for an hour.

**Figure 4.**
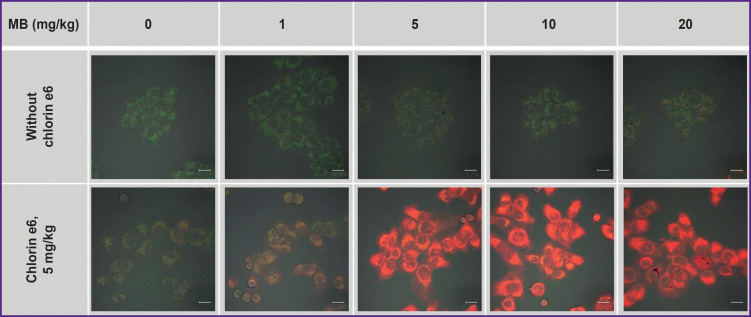
Fluorescence cell images incubated with MB and chlorin e6 Green pseudocolor corresponds to autofluorescence, red — to MB fluorescence. Bar — 20 μm

Cell incubation with MB only at the concentration up to 10 mg/kg does not increase essentially its fluorescence in the cells (Figure 4). Under such conditions, MB in the cells is assumed to transit to a photodynamically inactive leucoform. At the MB concentration of 20 mg/kg, separate cells with intensive uniform fluorescence are distinguished. This heterogeneity in the MB accumulation across the cell population may be interpreted as critical disturbance of membrane plasticity and membrane potential of the cell as a whole as the result of its greater activity in MB accumulation leading to aggregate formation.

**Figure 5.**
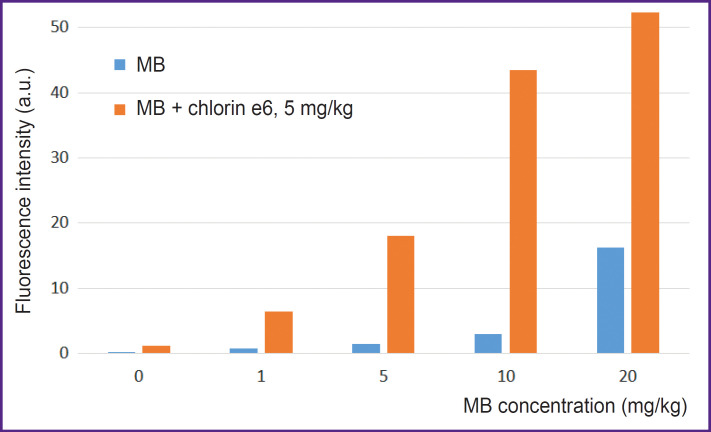
Integral fluorescence intensity of MB and chlorin e6 in the cells after 1 h of incubation of photosensitizers in the growth media

Cell incubation with MB concurrently with chlorin e6 leads to a great fluorescence of MB inside the cells relative to the cell incubation with MB alone (see Figure 4; Figure 5). Probably, chlorin e6 inhibits the cycle of tricarboxylic acids disrupting the reduction of MB to the leucoform. Besides, the decreased transition of MB to the leucoform may be related to a low-intensive irradiation during the scanning procedure, which results in ROS formation and reverse oxidation of the leucoform to MB.

### Photodynamic effect of MB

The HeLa cells were incubated with MB (1, 5, 10, and 20 mg/kg) and chlorin e6 (5 mg/kg) at 37°C, 5% CO_2_ for an hour. The following PDT parameters were used: 660 nm, 100 mW/cm^2^, 10 min, 60 J/cm^2^.

**Figure 6.**
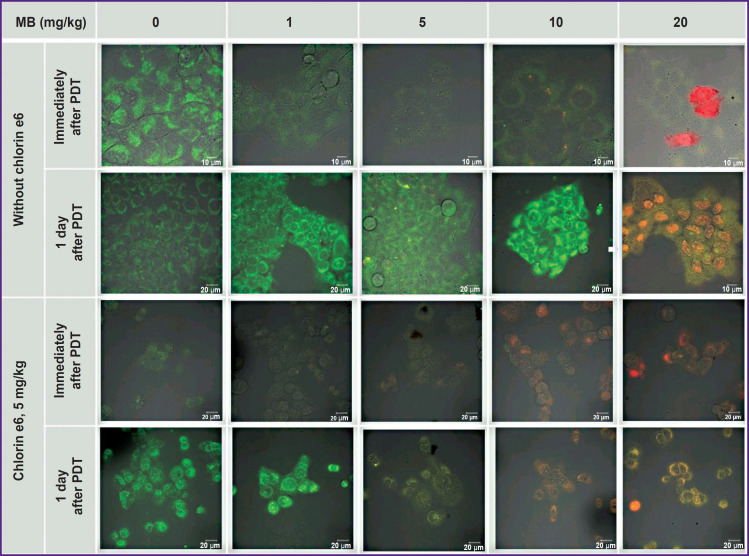
Fluorescence cell images incubated with MB and chlorin e6 immediately after PDT and in a day Green pseudololor corresponds to autofluorescence, red — to MB fluorescence

**Figure 7.**
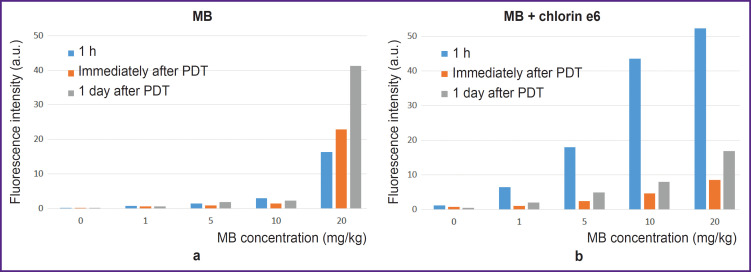
Integral MB fluorescence intensity (а) and MB and chlorin e6 (b) in the cells after 1 h of incubation of photosensitizers in the growth medium immediately after PDT and a day after PDT

All irradiated cells with MB and chlorin e6 have lost fluorescence in the red spectral region (Figures 6 and 7). Decreased MB fluorescence may be explained by photobleaching and also by the fact that PDT diminishes oxygen content leading to the transition of MB to the leucoform and inability of reverse oxidation to MB due to a low oxygen content.

The intensity of cell autofluorescence registered at excitation by 514 nm laser (flavins) indicated a powerful oxidative stress the day after irradiation. This phenomenon was especially pronounced at 1–10 mg/kg MB concentration and for 0–1 mg/kg and for the combination of chlorin e6 with MB. At the MB concentration of 20 mg/kg and for the combination of chlorin e6 with MB at 5–20 mg/kg, the oxidative stress is still greater resulting probably in a rapid necrosis since there is no time enough for the response to develop.

**Figure 8.**
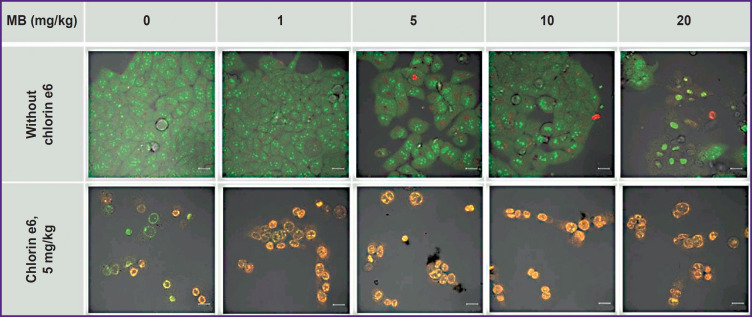
Fluorescence cell images a day after PDT with MB and chlorin e6 and MB stained with AO/PI Green pseudocolor — living cells, red — dead. Bar —20 μm

The MB concentration of 1–10 mg/kg and 60 J/cm^2^ laser radiation dose do not lead to cell death, probably due to the transition of MB to the photodynamically inactive leucoform. PDT with 20 mg/kg MB results in MB fluorescence in the cell nuclei in a day, while staining with AO/PI reveals PI nuclei staining, indicating cell death. The 60 J/cm^2^ dose causes cell death after PDT for all specimens with chlorin e6 and MB at 0–20 mg/kg (Figure 8). The intensity of staining the cell nuclei with PI after the exposure to PDT with MB and chlorin e6 illustrates a higher combined phototoxicity of MB and chlorin e6 relative to chlorin e6 alone.

### FLIM-phasor diagrams for the cells after the incubation with photosensitizers and after PDT

Fluorescence lifetime of the metabolic NADH cofactor has been analyzed after the incubation of the cells with MB and chlorin e6 and after PDT with them. Our interpretation of the cloud of points for NADH fluorescence lifetime in the phasor diagram relies on the existence of a “metabolic trajectory”, a conditional line between the 0.4 and 2.5 ns points, where the shorter lifetimes correspond to anaerobic glycolysis and the longer ones to oxidative phosphorylation [22, 23].

**Figure 9.**
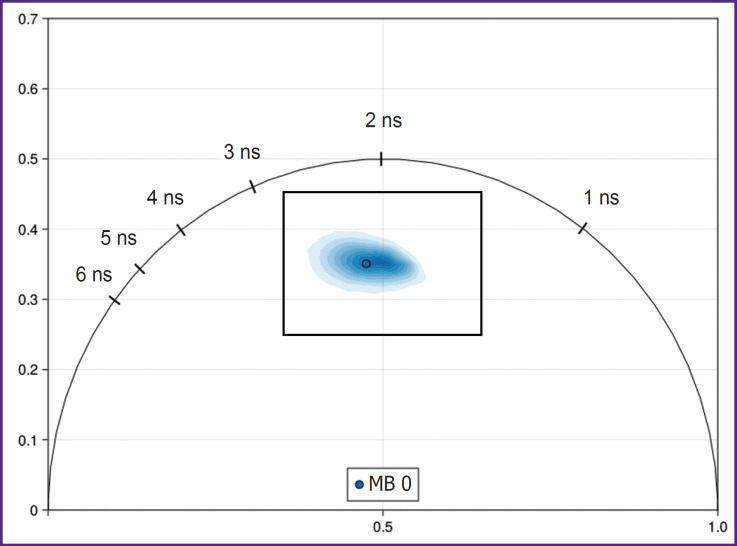
Phasor diagram of NADH fluorescence obtained for HeLa cell culture A box shows the diagram region interpreted further

Initially, the cloud of NADH fluorescence lifetime distribution for the HeLa cells under normal incubation conditions was in the center of the phasor diagram on the line connecting 0.5 and 3.4 ns (Figure 9).

A central region of the phasor diagram including the basic signal: 0.35–0.65 G (Х axis) and 0.25–0.45 S (Y axis) will be further shown for better representation of the changes (see Figure 9).

**Figure 10.**
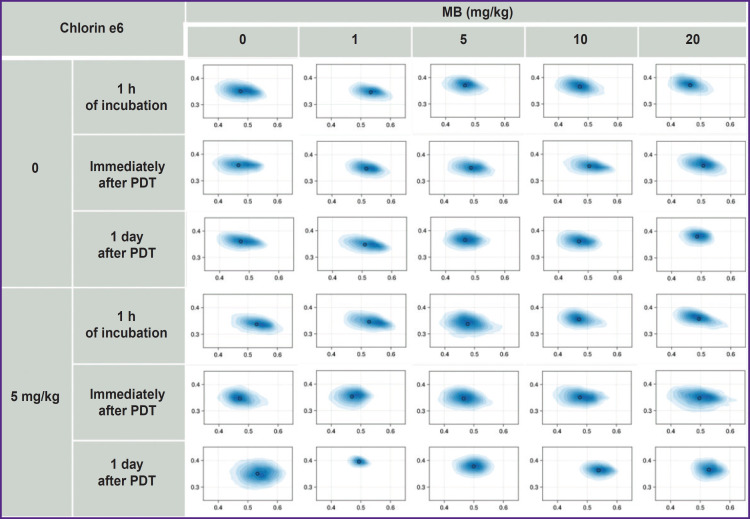
Phasor diagrams of NADH fluorescence, obtained for HeLa cell culture, incubated with MB and Mb with chlorin e6 for 1 h, immediately after and a day after photodynamic exposure

The cell stress from incubation with high MB concentrations (10– 20 mg/kg) and MB (1–20 mg/kg) simultaneously with chlorin e6 impairs the membrane potential in the first case and inhibits the cycle of tricarboxylic acids in the second, however, in the phasor diagram it is reflected as an insignificant extension of the NADH fluorescence cloud along the metabolic axis (Figure 10).

The photodynamic therapy depletes the oxygen level, which leads to the impairment of the normal cell metabolism. Thus, an insignificant level of hypoxia and oxidative stress was expected after PDT with low MB concentrations. In this case, the NADH fluorescence cloud shifts to the right in the phasor diagram towards the short lifetimes (closer to anaerobic glycolysis along the metabolic NADH trajectory).

However, acute hypoxia after PDT with MB and chlorin e6 shifts the NADH fluorescence cloud to the left in the phasor diagram towards the long lifetimes (closer to oxidative phosphorylation along the NADH trajectory), the cells die in this case due to necrosis. One day after PDT with the cell death, confirmed by staining with AO/ PI, rounding of the fluorescence cloud occurs in the phasor diagram with its small shift towards anaerobic glycolysis.

## Discussion

Application of photosensitizers for OXPHOS inhibition in order to diminish oxygen consumption by the tumor cells during the course of PDT is a promising therapeutic approach. The amphiphilic chlorin e6 is mainly bound to choline-containing head groups of phospholipid regions [24–26]. There are data of systematic study [27] of HeLa cell metabolic profile during dark period of incubation with chlorin photosensitizers using NMR spectroscopy. Thus, chlorin e4 has been established to cause inhibiting toxic effect on the tricarboxylic acid cycle and phosphatidylcholine metabolism. And the metabolic changes in the cells are interpreted as stimulation of defense mechanisms against non-physiological porphyrin xenobiotic.

The investigations of cytotoxic MB effects in the cells have shown the connection with mitochondrial damage. It has been demonstrated on mitochondrial suspensions that MB actively binds to mitochondria and penetrates into the matrix stimulated by the proton potential of mitochondria. However, a greater MB accumulation occurs in mitochondria with an increased proton potential, further it leads to the reduction of MB to photochemically inactive leucoform and MB dimer generation [28].

Oxidative stress and ROS play a central role in many physiological and pathophysiological processes. Metabolomic methods such as targeted liquid chromatography-tandem mass spectrometry (LCMS/ MS) are employed for quantitative estimation of specific metabolites including glutathione, which is directly involved in the redox homeostasis. Nontargeted metabolomics gives a comprehensive overview of metabolic changes caused by oxidative stress and can provide potential cancer biomarkers [29]. Correlation imaging allows for visualization of changes in metabolism (providing absolute quantities for >100 metabolites including the cycle of tricarboxylic acids, pentose phosphate pathway, purine metabolism, glutathione metabolism, metabolism of cysteine and methionine, glycolysis, and gluconeogenesis) at the concurrent microscopy of living cells [30]. Morphological and metabolic adaptation of cancer cells under a short exposure to hydrogen peroxide *in vitro* has been illustrated as an example. Application of metabolic inhibitors or determining enzyme activity by the addition of high-concentration substrates is one more method of metabolic profiling. There is a flow cytometry-based method for detailed analysis of metabolic profiling in heterogeneous cell populations called SCENITH [31]. On the other hand, there exists FLIM — a fast nondestructive method allowing for the study exogenous and endogenous fluorophores, which provides a high spatial and temporal resolution for imaging the cell types, cell cultures, and biopsies *in vitro* and *in vivo* [32].

We have investigated the fluorescence level of the endogenous NADH metabolite in a bound and free forms. As the absolute amount of the protein-bound NADH is relatively stable, the ratio of free (short lifetime) to protein-bound (long lifetime) NADH coincides with the NADH redox state, and so does the mean NADH lifetime. A shift of the cell metabolism towards glycolysis and/or decrease of mitochondrial respiration are accompanied by an increased NADH autofluorescence intensity and a shorter lifetime, which is reflected in a higher metabolic NADH index (a1/a2 ratio). Reverse changes denote the restored mitochondrial activity. Despite de-oxygenation, a strong oxidative stress and cell death occurring during the irradiation of the specimens with chlorin e6, great changes in the NADH fluorescence lifetime have not been registered. Probably, the cell must have time to rearrange the metabolite proportions to meet the changing conditions but there was no time to do it. For example, there are data showing that a long-lasting stress may cause the accumulation of lipid oxidation products fluorescing in the spectral NADH range with 7.8 ns fluorescence lifetime [33].

Besides, in hypoxia the cells alter the activity of NADH-bound enzymes, which must influence the changes in the long component of the NADH fluorescence lifetime. In hypoxia, the level of lactate dehydrogenase increases by more than 10 times relative to the normoxia conditions [34]. The fluorescence lifetime for lactate dehydrogenase-bound NADH is 1.6 ns [35]. The extension of the fluorescence cloud in the phasor diagram towards the lactate dehydrogenase-bound NADH (1.6 ns) on the one hand, and towards the lipid oxidation products (7.8 ns) on the other, should have turned the cloud transversely to the metabolic trajectory, but it was not observed in our experiments.

Nevertheless, the accumulation of MB together with chlorin e6 by the cells results in the intracellular alterations, expressed in the MC capacity to reduce to the leucoform, while subsequent irradiation causes more rapid cell death if compared to the use of a photosensitizer alone.

## Conclusion

The effect of photosensitizers MB and MB in combination with chlorin e6 on cell metabolism in case of their co-accumulation and subsequent irradiation has been studied. The idea of their combined application consisted in the inhibiting the cycle of tricarboxylic acids with chlorin e6 leading to OXPHOS inhibition. In this case, the oxygen uptake by the cells decreases and reduction of MB to the colorless leucoform is disrupted, which allows for concurrent activation of type I and II photodynamic reactions. We have proved that coaccumulation decreases the rate of MB transition to the colorless photoinactive leucoform and the effect of type I PDT from MB increases supplementing the effect from type II PDT with chlorin e6.
